# Chemotherapy is associated with faster abdominal aortic aneurysm growth in patients with concomitant cancer in a multicenter cohort

**DOI:** 10.1016/j.clinsp.2026.101099

**Published:** 2026-09-06

**Authors:** Harue Santiago Kumakura, Andressa Cristina Sposato Louzada, Glauco Fernandes Saes, Guilherme Yazbek, Nelson De Luccia, Pedro Puech-Leão, Nelson Wolosker, Antonio Eduardo Zerati

**Affiliations:** a Hospital das Clínicas da Faculdade de Medicina da Universidade de São Paulo (HCFMUSP), São Paulo, SP, Brazil; b Hospital Israelita Albert Einstein (HIAE), São Paulo, SP, Brazil; c Faculdade Israelita de Ciências da Saúde Albert Einstein, São Paulo, SP, Brazil; d Instituto do Cancer do Estado de São Paulo (ICESP), São Paulo, SP, Brazil; e A. C. Camargo Cancer Center (ACCCC), São Paulo, SP, Brazil; f Faculdade de Medicina da Universidade de São Paulo (FMUSP), São Paulo, SP, Brazil

**Keywords:** Cancer treatments, Radiotherapy, Aortic Aneurysm growth rate, Chemotherapy drugs

## Abstract

**Objective:**

To investigate the prevalence of synchronous Abdominal Aortic Aneurysm (AAA) and malignancy and the impact of different cancer treatments on the natural history of aortic aneurysms, focusing on its growth and risk of rupture.

**Design:**

Retrospective multicenter observational study.

**Methods:**

Imaging scans were examined to select patients with abdominal aortic aneurysms. Aortic diameters, aortic rupture, demographic and clinical data from patients with abdominal aortic aneurysms with or without cancer (controls) treated at four medical centers in Brazil were retrospectively analyzed.

**Results:**

27,455 abdominal scans were screened, resulting in 297 patients with AAA being included. Of these patients, 220 had concomitant cancer. The prevalence of AAA and cancer was 2.4%. A further 73 patients with AAA but no cancer were recruited from an outpatient cohort to also join the control group of 77 patients, resulting in 150. No aortic rupture was observed. In multivariable analysis, larger baseline aortic diameter (p < 0.001) and exposure to alkylating agents (p = 0.023), antimetabolites (p = 0.023), and topoisomerase inhibitors (p = 0.003) were independently associated with increased AAA growth rates. Neither cancer diagnosis nor radiotherapy exposure was significantly associated with AAA growth.

**Conclusions:**

2.4% of cancer patients had a concomitant AAA. Aneurysm growth rates were significantly influenced by the initial aortic diameter and by treatment with three classes of chemotherapy drugs: alkylating agents, antimetabolites and topoisomerase inhibitors.

## Highlights

• The prevalence of abdominal aortic aneurysm was 2.4% in patients with cancer.
• Alkylating agents, antimetabolites and topoisomerase inhibitors had higher aneurysm growth rates.
• Having cancer or undergoing radiotherapy did not impact the natural history of AAA.

## Introduction

The prevalence of both Abdominal Aortic Aneurysms (AAA) and cancer is expected to increase as the elderly population grows. The association of these two conditions is also expected to increase, not only because they share some risk factors, such as advanced age and smoking, but also because cancer patients frequently undergo more imaging tests, raising the likelihood of incidental diagnosis.

The association between AAA and cancer is estimated at 1%‒17% of patients with AAA^1^ However, the impact of cancer and its treatments on the growth and rupture of AAA remains controversial^1^, ^2^, ^3^, ^4^ This controversy poses a significant challenge to physicians, as there are no specific guidelines on which life-threatening condition should be addressed first. While common sense may suggests staged treatments, prioritizing the condition that poses the greatest or most immediate risk to the patient, there are also reports of safe concurrent treatment^5^^,^^6^

Although there are studies suggesting no difference in AAA growth rates in patients with AAA and cancer who have received Chemotherapy (ChT),^3^ some other authors have reported faster growth and a greater risk of rupture in this population^1^^,^^7^^,^^8^

More recently, larger cohorts have helped to identify that some specific cancer treatments may impact the natural history of AAA. Among the various chemotherapeutic agents, antimetabolites have been shown to increase AAA growth rates^2^^,^^4^ Additionally, another study has reported that Radiotherapy (RT) reduced the growth of AAA^4^

Most of these studies focused mainly on oncologic patients from single-center cohorts in Europe and the United States of America. However, it is important to note that AAA repair and mortality rates can differ based on location and socioeconomic status^9^^,^^10^ Even though a recent report indicated that Latin America has the highest age-standardized death rate related to AAA,^11^ there are currently no studies examining the impact of cancer, chemotherapy and radiotherapy on AAA in this population.

We conducted a multicenter study involving Brazilian cohorts to investigate the prevalence of AAA and cancer. We also investigated the impact of different cancer treatments on AAA progression, specifically focusing on its growth and risk of rupture.

## Materials and methods

### Patient selection

Imaging scans, demographic and clinical data from patients with AAA treated at four medical centers in Brazil were retrospectively analyzed. The primary outcome was AAA growth. A vascular surgeon reviewed all scans to confirm the aortic diameter. The study was designed and reported in accordance with the Strengthening the Reporting of Observational Studies in Epidemiology (STROBE) guidelines^12^

The inclusion criteria were an abdominal aortic diameter of at least 3 cm and a minimum of two multi-planar Computed Tomography (CT) scans obtained at an interval of at least six months, with at least one of the scans using intravenous contrast to rule out dilated dissections. The exclusion criteria were patients under 18-years of age, patients with incomplete clinical documentation or imaging studies, and patients with previous aortic treatments.

The sampling strategies varied among the participating centers: at Instituto do Cancer do Estado de São Paulo (ICESP), all 12,772 abdominal CT scans performed between 2008 and 2017 were screened to identify AAA in patients followed at the oncology center. At A. C. Camargo Cancer Center (ACCCC), abdominal CT scans performed between 2008 and 2021 were screened using a full-text query containing the keywords “aneurysm” and “aortic”. At Hospital Israelita Albert Einstein (HIAE), abdominal CT scans obtained between 2007 and 2017 were also screened using the same full-text query strategy. Medical records of oncologic and non-oncologic patients with confirmed AAA were subsequently reviewed. At Hospital das Clínicas da Faculdade de Medicina da Universidade de São Paulo (HCFMUSP), consecutive non-cancer patients undergoing routine surveillance for small AAA between 2008 and 2017 were included as a convenience control cohort. These patients fulfilled the same inclusion criteria applied to the cancer cohort, including an aortic diameter ≥ 3 cm and at least two CT scans obtained at a minimum interval of six months.

### Image analyses

All aortic diameters on CTs were double-checked by a vascular surgeon using Intuition software (OsiriX, Geneva, Switzerland)^13^ For cancer patients, the diameter was recorded in the initial CT scans before oncologic treatment and in the last CT after oncologic treatment. The diameters in the first and last available CTs were used for non-cancer patients. AAA growth rates were calculated as the difference between the initial and final aortic diameters divided by the time interval between the CTs and expressed in millimeters per year (mm/year). Based on expected AAA growth rates according to baseline aneurysm diameter reported by Powell et al.,^14^ patients presenting disproportionately elevated aneurysm growth rates were considered outliers.

### Clinical data

Patient demographics, comorbidities, type of cancer and oncologic treatment details (chemotherapy agents, radiotherapy and surgery) were gathered from the medical records retrospectively.

### Statistical analysis

Categorical data were presented as frequencies and compared using the chi-squared test. Based on their distribution, numerical data were expressed as mean and standard deviation or median and 95% Confidence Interval. This study investigated the effects of demographic and clinical data, as well as cancer treatment, on AAA growth rates using Kruskal-Wallis. Multivariable linear regression models were used to assess the independent association between exposure to different chemotherapeutic classes and AAA growth rate, with adjustment for baseline AAA diameter, sex, and smoking status. Effect estimates were expressed as adjusted β-coefficients and corresponding 95% Confidence Intervals (95% CI).

Statistical analyses were performed using IBM-SPSS for Windows software version 20.0, and with a significance level of 5%.

### Ethics

This study was approved by the Ethics Committee of the four participating centers (CAAE: 31,354,714.1.3002.0071). Since thousands of CT scans were screened and data were obtained retrospectively, the Institutional Review Boards granted a waiver of informed consent forms.

## Results

**Fig. 1:**
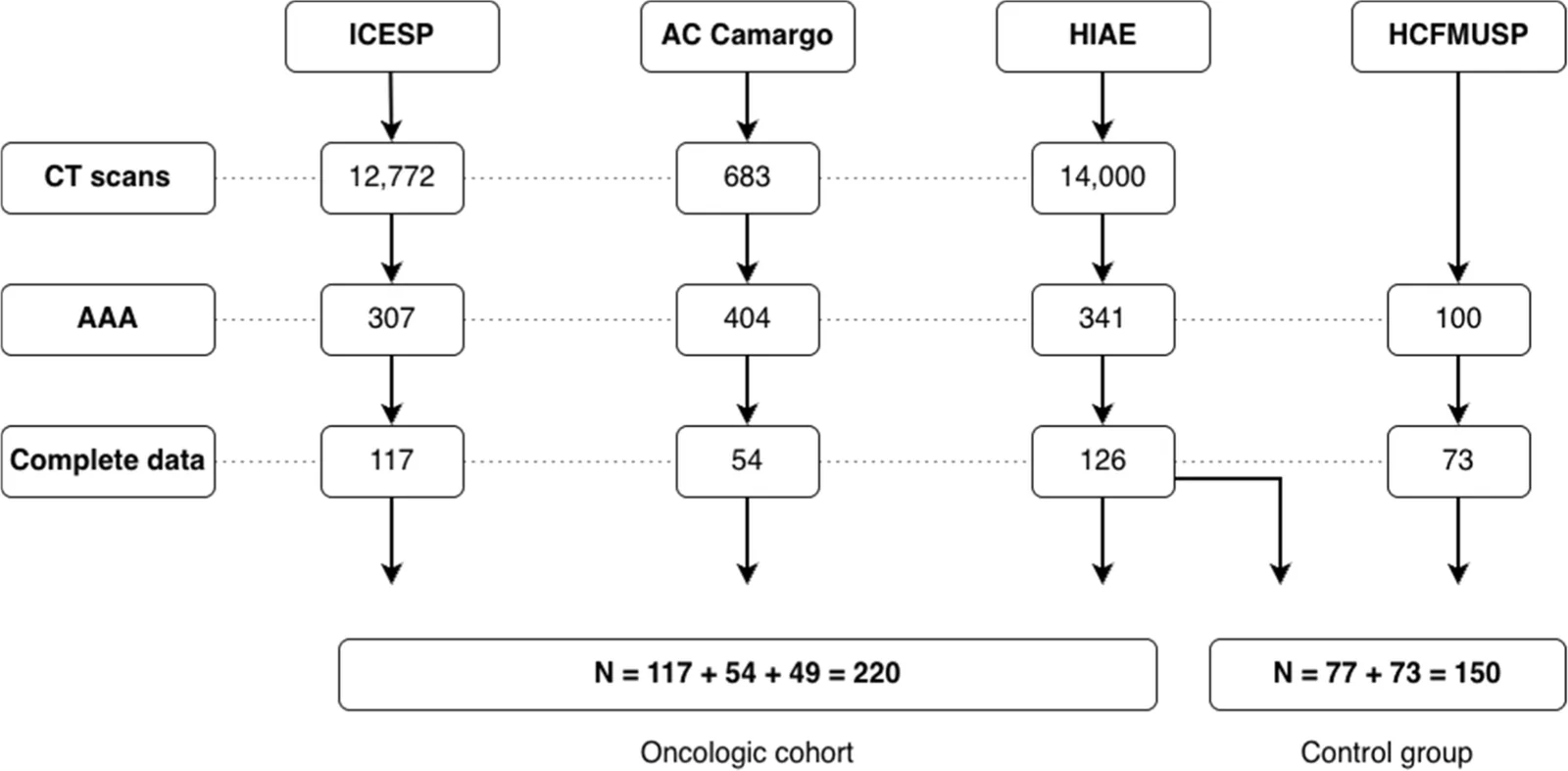
Flowchart of patient selection and cohort allocation across the participating centers. Footnotes: ICESP, Instituto do Cancer do Estado de São Paulo; HIAE, Hospital Israelita Albert Einstein; HCFMUSP, Hospital das Clínicas da Faculdade de Medicina da Universidade de São Paulo; CT, computed tomography; AAA, abdominal aortic aneurysm.

After reviewing 27,455 abdominal CT scans, 297 patients from ICESP, ACCCC, and HIAE were included, 220 of which had concomitant cancer. Additionally, 73 consecutive non-cancer outpatients under routine surveillance for small AAA at HCFMUSP were enrolled to the control group of 77 patients, resulting in 150 (Fig. 1).

The mean age was 71.7 years old, with the majority being male (78.1%) and having a history of smoking (23.5% current smokers, 43.5% former smokers) and hypertension (66.5%). In addition, 37% of the patients had dyslipidemia, and 24.3% had diabetes mellitus.

A total of 220 patients (59.5%) had both cancer and AAA. These patients were categorized into 4 groups based on their oncological treatment: 59 (26.8%) received no Chemotherapy or Radiotherapy (No ChT/No RT), 75 (34.1%) received Chemotherapy but No Radiotherapy (ChT/No RT), 27 (12.3%) received Radiotherapy but No Chemotherapy (RT/No ChT) and 59 patients (26.8%) were treated with both Chemotherapy and Radiotherapy (Cht+RT).

At the ICESP oncologic center, where all abdominal CT scans were analyzed, we observed a 2.4% prevalence of association between AAA and cancer.

The clinical and demographic data for the non-cancer control group and cancer treatment groups are summarized in Table 1. We found that there were significant differences between the groups in terms of hypertension (p = 0.047), smoking status (p = 0.009), and age (p < 0.001). Further analysis revealed that patients in No ChT/No RT group were significantly older than those in the Control group (p = 0.008), ChT/No RT (p = 0.042), and ChT+ RT (p = 0.002).

**Table 1:** Summary of patients’ demographic and clinical data according to treatment groups.

	No ChT/ No RT	ChT/ No RT	RT/ No ChT	Cht+RT	Control	p
**Age (mean, years)**	74.7	70.8	74.1	68.8	70.5	<0.001^a^
**Sex (% Male)**	81.3	82.6	77.8	86.4	71.3	0.07^b^
**SAH (%)**	76.3	60	85.2	59.3	65.3	0.047^b^
**DM (%)**	22	24	33	18.6	26	0.622^b^
**DLP (%)**	44.1	75	44.4	23.7	40.7	0.09^b^
**Smoking status (%)**	71.2	72	70.4	84.7	55.3	0.009^b^

Footnotes: SAH, Systemic Arterial Hypertension; DM, Diabetes Mellitus; DLP, Dyslipidemia. Smoking status: current and former smokers; Chemotherapy or Radiotherapy (No ChT/No RT), Chemotherapy but No Radiotherapy (ChT/No RT), Radiotherapy but No Chemotherapy (RT/No ChT), both Chemotherapy and Radiotherapy (Cht+RT).

a ANOVA. ^b^ Chi-Squared test.

The types of cancer most frequently observed in patients with AAA were colorectal cancer (27.3%), lung cancer (17.3%) and prostate cancer (12.7%).

Data on aortic diameters are presented in Table 2. The overall mean initial aortic diameter was 39.5 ± 7.8 mm (range 30–74 mm), with no statistically significant difference between the groups.

**Table 2:** Initial aortic diameter according to treatment groups.

	Initial Diameter	p-value
**No ChT/No RT**	40.9 ± 8.5	0.197^a^
**ChT/No RT**	38.4 ± 6.8	
**RT/No ChT**	38.2 ± 9.5	
**Cht+RT**	39.2 ± 8.7	
**Control**	39.2 ± 7.5	

Footnotes: Chemotherapy or Radiotherapy (No ChT/No RT), Chemotherapy but No Radiotherapy (ChT/No RT), Radiotherapy but No Chemotherapy (RT/No ChT), both Chemotherapy and Radiotherapy (Cht+RT).

a Kruskal-Wallis test.

Table 3 details the growth rates among the groups. The overall mean value was 2.33±2.24 mm/year (range 0–24 mm/year), and we did not observe a statistically significant difference between the groups (p = 0.706).

**Table 3:** Comparison of AAA growth rates between groups.

Growth rate (mm/year)	Median (95% CI)	Mean ± SD	p-value
No ChT/No RT	2.00 (1.70‒2.90)	2.48 ± 7.20	0.706^a^
ChT/No RT	1.90 (1.50‒2.55)	2.31 ± 10.0	
RT/No ChT	1.37 (1.10‒2.40)	1.84 ± 8.60	
Cht+RT	1.95 (1.42‒2.40)	2.63 ± 24.0	
Control	1.70 (1.50–2.10)	2.24 ± 15.24	

Footnotes: Chemotherapy or Radiotherapy (No ChT/No RT), Chemotherapy but No Radiotherapy (ChT/No RT), Radiotherapy but No Chemotherapy (RT/No ChT), both Chemotherapy and Radiotherapy (Cht+RT); CI, Confidence Interval; SD, Standard Deviation.

a Mann-Whitney test.

Based on expected AAA growth rates according to baseline aneurysm diameter described in the literature,^13^ 15 patients were identified as outliers due to disproportionately elevated aneurysm growth rates. Among these patients, 9 (60%) had concomitant cancer, including gastrointestinal (n = 3), lung (n = 2), bladder (n = 2), and hematologic malignancies (n = 1). Two patients underwent radiotherapy and six received different chemotherapy regimens. No distinct clinical or treatment-related pattern was identified among these cases.

Sub-analyses of the factors that might have affected the growth rates of AAA, showed that the initial diameter was significantly positively correlated with growth rates (p < 0.001). Additionally, demographic characteristics and comorbidities, including type of cancer and general oncologic treatments, did not appear to have an impact on the growth rate.

To further evaluate the association between chemotherapy exposure and AAA growth rate, multivariable linear regression analyses were performed adjusting for baseline AAA diameter, sex, and smoking status. Each chemotherapeutic class was analyzed separately (Table 4). Exposure to alkylating agents (adjusted β = 0.682, 95% CI 0.095–1.269; p = 0.023), antimetabolites (adjusted β = 0.694, 95% CI 0.096–1.293; p = 0.023), and topoisomerase inhibitors (adjusted β = 1.262, 95% CI 0.421–2.102; p = 0.003) remained independently associated with increased AAA growth rates after adjustment for potential confounders.

**Table 4:** Adjusted β-coefficients derived from a multivariable linear regression model adjusted for baseline aneurysm diameter, sex, and smoking status.

Chemotherapeutic agent	Adjusted β-coefficient	(95% CI)	p-value
Alkylating agents	0.682	(0.095 – 1.269)	0.023
Antimetabolites	0.694	(0.096 – 1.293)	0.023
Topoisomerase inhibitors	1.262	(0.421 – 2.102)	0.003
Alkaloids	−0.338	(−1.025 – 0.349)	0.334
Cytotoxic antibiotics	−0.429	(−1.630 – 0.773)	0.483
Monoclonal antibodies	0.413	(−0.432 – 1.258)	0.336
Topoisomerase inhibitors	−0.259	(−1.798 – 1.280)	0.741
Corticoid	−0.093	(−1.740 – 1.553)	0.911
Hormonal blockade	−0.289	(−1.472 – 0.893)	0.630

CI, Confidence Interval.

The mean follow-up duration between CT scans was 2,9 years for cancer group e 3,4 years for control group**.** No aortic rupture was identified in the CT scans or reported in the medical records.

## Discussion

To the best of our knowledge, this study includes the largest cohort of patients with synchronous AAA and cancer in the literature and it is the only one conducted in Latin America, where the burden of AAA is high^15^

Our search for AAA in abdominal CT scans in cancer patients showed that 2.4% had AAA. This prevalence falls within the range reported in the literature,^1^ highlighting the relevance of studying the impact of cancer and its treatments on the evolution of AAA.

The clinical and demographic data of our cohort were similar to those reported by other authors^16^^,^^17^ Although there were differences between the groups in terms of hypertension, smoking, and age, our analyses showed that none of these factors influenced the growth rates of AAA we observed.

In our study, the presentation of concomitant malignancy did not appear to affect the natural history of AAA, which is in line with the findings of Maxwell et al.,^2^ but contrasts with the results of the study by Becker von Rose et al.,^4^ who observed that patients with renal cell carcinoma presented a significantly lower rate of AAA growth. Even though our cohort seemed to have a similar proportion of patients with both renal cell carcinoma and AAA to theirs (approximately 4% in both studies), we did not observe any significant difference. Further studies are needed to better evaluate this correlation.

There was also no significant difference in the growth rates between the control group and the groups that received chemotherapy, consonant with other large studies^2^^,^^4^

Notably, in the subgroup analysis evaluating the impact of different chemotherapy classes, we found that alkylating agents, antimetabolites, and topoisomerase inhibitors were significantly associated with increased AAA growth rates. Importantly, these associations remained significant after multivariable adjustment for baseline AAA diameter, sex, and smoking status, suggesting that the observed effects were independent of these established factors related to aneurysm progression. Other researchers have also reported similar findings regarding antimetabolites and topoisomerase inhibitors,^2^^,^^4^ but we also observed an increase in AAA growth rates after treatment with alkylating agents. The biological mechanisms underlying this association remain incompletely understood. However, AAA progression is known to involve chronic vascular inflammation, extracellular matrix degradation, oxidative stress, and apoptosis of vascular smooth muscle cells^14^ Chemotherapeutic agents may potentially exacerbate these pathways through endothelial injury, increased inflammatory cytokine release, activation of matrix metalloproteinases, and impaired tissue repair mechanisms^2^^,^^4^ Alkylating agents, in particular, exert non-specific cytotoxic effects across multiple phases of the cell cycle and may therefore contribute to vascular wall degeneration in biologically active aortic tissue. Nevertheless, these hypotheses remain largely mechanistic and speculative, as direct evidence linking specific chemotherapeutic agents to AAA progression is still limited. Further experimental and prospective clinical studies are needed to better understand the biological impact of chemotherapy on aneurysm behavior.

Research has shown that radiotherapy can cause inflammation in the diseased vessels^18^ When focusing on the patients with AAA treated with radiotherapy, Becker et al. ^4^ found a significant decrease in AAA growth rates. Although our study observed the lowest median AAA growth rates among patients treated with radiotherapy alone, no statistically significant association between radiotherapy exposure and AAA growth rates was identified in this cohort. However, these findings should be interpreted cautiously due to the relatively small radiotherapy subgroup and the absence of detailed radiation field and dosimetric data, which prevented confirmation of direct aneurysm radiation exposure.

We did not observe any AAA ruptures during the study period. This finding may be related to the predominance of small aneurysms in the cohort, adherence to surveillance protocols with repair at guideline-recommended thresholds,^17^ and the duration of imaging follow-up. Although accelerated aneurysm growth may indicate altered aneurysm biology and potentially increased future risk, our study was not designed to assess long-term clinical outcomes such as rupture or aneurysm-related mortality.

Based on our results, we do not recommend treating small AAA in patients with cancer just because of this association. However, cancer patients undergoing chemotherapy with alkylating agents, antimetabolites, or topoisomerase inhibitors may benefit from personalized imaging surveillance, as they seem to experience faster AAA growth. Nevertheless, additional prospective studies are required to confirm this finding.

This study has several limitations, including the retrospective design, including patients' post-CT scans, and the absence of details regarding the radiation plan. Another limitation is the use of a non-matched convenience sample as the control group, which may have introduced selection bias. Although the same imaging eligibility criteria were applied to both cohorts, residual differences between oncologic and non-oncologic patients cannot be excluded. However, it stands as the largest cohort examining concomitant AAA and malignancy. The study is unique in that it is the only one conducted in Latin America. It is also important because it showed how much of an effect alkylating agents’ treatment may have on the growth rate of AAA.

## Conclusions

The occurrence of synchronous AAA and malignancy was found to be at 2.4%. The results from this study suggest that the growth rates of AAA were significantly influenced by the initial AAA diameter and treatment with three classes of chemotherapy drugs: alkylating agents, antimetabolites, and topoisomerase inhibitors. In this cohort, no statistically significant association was observed between radiotherapy exposure and AAA growth rates. However, interpretation of this finding is limited by the small number of patients treated with radiotherapy alone and by the absence of detailed radiation field data.

## Data availability

Data are available from the corresponding author upon reasonable request.

## Funding

This research did not receive any specific grant from funding agencies in the public, commercial, or not-for-profit sectors.

## CRediT authorship contribution statement

**Harue Santiago Kumakura:** Conceptualization, Methodology, Investigation, Data curation, Writing – original draft, Writing – review & editing. **Andressa Cristina Sposato Louzada:** Conceptualization, Methodology, Investigation, Data curation, Writing – original draft, Writing – review & editing. **Glauco Fernandes Saes:** Conceptualization, Methodology, Investigation, Data curation, Writing – review & editing. **Guilherme Yazbek:** Conceptualization, Methodology, Writing – review & editing. **Nelson De Luccia:** Conceptualization, Methodology, Writing – review & editing. **Pedro Puech-Leão:** Conceptualization, Methodology, Writing – review & editing. **Nelson Wolosker:** Conceptualization, Methodology, Writing – review & editing. **Antonio Eduardo Zerati:** Conceptualization, Methodology, Investigation, Data curation, Supervision, Writing – original draft, Writing – review & editing.

## Conflicts of interest

The authors declare no conflicts of interest.
